# *Paenibacillus larvae* Chitin-Degrading Protein *Pl*CBP49 Is a Key Virulence Factor in American Foulbrood of Honey Bees

**DOI:** 10.1371/journal.ppat.1004284

**Published:** 2014-07-31

**Authors:** Eva Garcia-Gonzalez, Lena Poppinga, Anne Fünfhaus, Gillian Hertlein, Kati Hedtke, Agata Jakubowska, Elke Genersch

**Affiliations:** 1 Institute for Bee Research, Department for Molecular Microbiology and Bee Diseases, Hohen Neuendorf, Germany; 2 Humboldt Universität Berlin, Institut für Biologie, Berlin, Germany; 3 Department of Genetics, Universitat de València, Burjassot, Spain; 4 Freie Universität Berlin, Institute of Microbiology and Epizootics, Berlin, Germany; Stanford University, United States of America

## Abstract

*Paenibacillus larvae*, the etiological agent of the globally occurring epizootic American Foulbrood (AFB) of honey bees, causes intestinal infections in honey bee larvae which develop into systemic infections inevitably leading to larval death. Massive brood mortality might eventually lead to collapse of the entire colony. Molecular mechanisms of host-microbe interactions in this system and of differences in virulence between *P. larvae* genotypes are poorly understood. Recently, it was demonstrated that the degradation of the peritrophic matrix lining the midgut epithelium is a key step in pathogenesis of *P. larvae* infections. Here, we present the isolation and identification of *Pl*CBP49, a modular, chitin-degrading protein of *P. larvae* and demonstrate that this enzyme is crucial for the degradation of the larval peritrophic matrix during infection. *Pl*CBP49 contains a module belonging to the auxiliary activity 10 (AA10, formerly CBM33) family of lytic polysaccharide monooxygenases (LPMOs) which are able to degrade recalcitrant polysaccharides. Using chitin-affinity purified *Pl*CBP49, we provide evidence that *Pl*CBP49 degrades chitin via a metal ion-dependent, oxidative mechanism, as already described for members of the AA10 family. Using *P. larvae* mutants lacking *Pl*CBP49 expression, we analyzed *in vivo* biological functions of *Pl*CBP49. In the absence of *Pl*CBP49 expression, peritrophic matrix degradation was markedly reduced and *P. larvae* virulence was nearly abolished. This indicated that *Pl*CBP49 is a key virulence factor for the species *P. larvae*. The identification of the functional role of *Pl*CBP49 in AFB pathogenesis broadens our understanding of this important family of chitin-binding and -degrading proteins, especially in those bacteria that can also act as entomopathogens.

## Author Summary

American Foulbrood and its etiological agent, *Paenibacillus larvae*, pose a serious threat to global honey bee health. So far, molecular mechanisms of host-microbe interactions are poorly understood in this system and no key virulence factor for the entire species has been identified. Here, we demonstrate that *P. larvae* expresses a chitin-binding and -degrading protein *Pl*CBP49 harboring one module that belongs to the auxiliary activity 10 (AA10) family of lytic polysaccharide monooxygenases (LPMOs). We provide evidence that *Pl*CBP49 degrades chitin via a metal ion-dependent, oxidative mechanism, as already described for other members of the AA10 enzyme family. Using *P. larvae* mutants lacking *Pl*CBP49 expression, we demonstrate that *Pl*CBP49 is crucial for the degradation of the chitin-rich peritrophic matrix, a key step in pathogenesis of *P. larvae* infections. In the absence of *Pl*CBP49 expression the peritrophic matrix remained nearly intact and about 95% of the infected larvae survived infection. This clearly indicated that *Pl*CBP49 is a key virulence factor of *P. larvae*. These results constitute important progress in our understanding of both *P. larvae* pathogenesis and the biological role of LPMOs in entomopathogens. Furthermore, knowing *Pl*CBP49 and its role in pathogenesis opens new possibilities to develop curative measures for this disease.

## Introduction

Vertebrates and invertebrates alike need to protect their intestinal epithelia against various chemical, physical and biological challenges while the transport of nutrients and water must remain uninterrupted to aid in digestion. For this purpose, mucosal secretions line the digestive tract of vertebrates, while in most invertebrates the midgut epithelium is lined by the peritrophic membrane or peritrophic matrix (PM) [1], [2], an organized layer made of secreted chitin and (glyco)proteins. Chitin, an insoluble linear β-(1,4)-linked polymer of *N*-acetyl-D-glucosamine (GlcNAc), is considered the major structural component of the PM where it forms chitin fibrils. These fibrils are held together by chitin-binding proteins, while the interstitial spaces are filled by glycans (for a recent review see [3]). The resulting lattice acts as a molecular sieve with a large range of pore sizes. The functions attributed to the PM include (i) compartmentalization of digestive processes, (ii) protection from ingested xenobiotics, and (iii) acting as mechanical barrier against abrasive foodstuffs and pathogens (for a recent review see [2]). The latter function makes the PM a first-line defense against ingested pathogens and, hence, an important part of the invertebrates' complex system to combat infections. Accordingly, insect-pathogenic bacteria infectious *per os* must breach the PM before they can attack and invade or cross the epithelium. To this end, hydrolytic enzymes such as proteases and, most importantly, chitin-degrading proteins enabling PM degradation are produced and secreted by these pathogens.

Most of the chitin-degrading proteins are chitinases belonging to the family of glycosyl hydrolases (GH). To date, 133 different GH families classified on the basis of sequence similarities and forming 14 clans (GH-A – GH-N) of related families based on similarities in protein folds can be found in the Carbohydrate Active Enzymes (CAZy) database [4]. Another family of bacterial chitin-degrading proteins comprises proteins with a carbohydrate-binding module (CBM) and belong to the auxiliary activities 10 (AA10) family (formerly chitin binding module 33 (CBM33) family) of lytic polysaccharide monooxygenases (LPMOs) as defined in the Carbohydrate Active Enzymes (CAZy) database [4]. These proteins were originally thought to lack any catalytic activity of their own [4], although they had been shown to be involved in chitin degradation [5]. However, it was recently demonstrated that CBP21, an AA10 (formerly CBM33) family member expressed by the Gram-negative soil bacterium *Serratia marcescens* [6], [7], as well as *Ef*CBM33A, expressed by the Gram-positive, opportunistic pathogen *Enterococcus faecalis*, are capable of degrading crystalline chitin via a novel, copper-dependent, oxidative enzymatic mechanism [8]–[10]. LPMOs comprise only two families, (i) the AA10 (formerly CBM33) family with bacterial, viral, and some eukaryotic members and (ii) the AA9 (formerly glycoside hydrolase 61 (GH61)) family with only fungal members so far. Both families are monooxygenases and target recalcitrant polysaccharides.

Bacterial chitin-degrading proteins are produced mainly to meet nutritional needs of the bacteria because chitin-degradation products, once transported into the bacterial cell, can be used as carbon sources [11], [12]. Because, in addition, many insect pathogens need to overcome chitin-containing structures (e.g., cuticula or peritrophic membranes) to enter a host and establish an infection, degradation of the PM by bacterial pathogens might be a process serving two purposes: nutrition and invasion.

*Paenibacillus larvae* is a bacterial pathogen of honey bee larvae which causes a notifiable disease called American Foulbrood (AFB) [13]. AFB is a highly contagious disease and is fatal for the entire colony when detected in too late a stage of disease. However, since most authorities consider burning of diseased colonies and contaminated hive material the only workable control measure, diseased colonies are inevitably lost in most cases. Hence, AFB causes considerable economic losses in apiculture worldwide. The etiological agent, *P. larvae*, is a Gram-positive, rod-shaped bacterium forming tenacious spores under adverse environmental conditions like lack of nutrients. These spores are the infectious form of *P. larvae* and they initiate a fatal infectious process in bee larvae once ingested with contaminated larval food. Honey bee larvae become less susceptible to infection with increasing age and as soon as two days after egg hatching they can be considered “resistant” (for recent reviews: [14]–[16]). This phenomenon has been attributed to the growing PM already in the early days of AFB research [17], [18]. Recently, we demonstrated that the honey bee larval gut is lined by a chitin-containing PM which is degraded during *P. larvae* infection [19], confirming earlier results showing that the PM is the first barrier the bacteria have to overcome when trying to breach the epithelium and to enter the haemocoel [20]. Proteases and chitin-degrading proteins are most likely involved in this process. For *P. larvae* it is long since known that an impressive number of proteases is expressed and secreted [21], [22]. These proteases, although poorly characterized, have already been implicated as virulence factors as they might aid in degrading the PM, breaching the epithelium and converting larval into bacterial biomass [23]–[25]. In contrast, little is known so far about the nature and expression of *P. larvae* chitin-degrading proteins and their role in the pathogenesis of AFB. Recently, the genomes of representatives of two *P. larvae* genotypes, ERIC I and ERIC II, were sequenced, annotated and used for comparative genome analysis [26]. Surprisingly, no complete, uninterrupted and, hence, putatively functional gene coding for a classical chitinase could be detected in any of the genomes [26] posing the intriguing question: how is the described chitin-degradation by *P. larvae* during infection [19] achieved? We are now answering this question by describing the identification and functional characterization of *P. larvae Pl*CBP49, a novel member of the AA10 (formerly CBM33) family of chitin-binding and –degrading LPMOs. *Pl*CBP49 was confirmed in the genome and secretome of *P. larvae*. Chitin-affinity purified *Pl*CBP49 was used to demonstrate chitinolytic activity both on soluble and insoluble substrates as well as to confirm that chitin degradation was metal ion-dependent and involved an oxidative step. Furthermore, we studied the biological role of *Pl*CBP49 in *P. larvae* infected larvae and were able to demonstrate that *Pl*CBP49 is involved in PM degradation during infection and is a key virulence factor of *P. larvae*.

## Results

### *P. larvae* expresses chitin-binding and -degrading proteins

**Figure 1:**
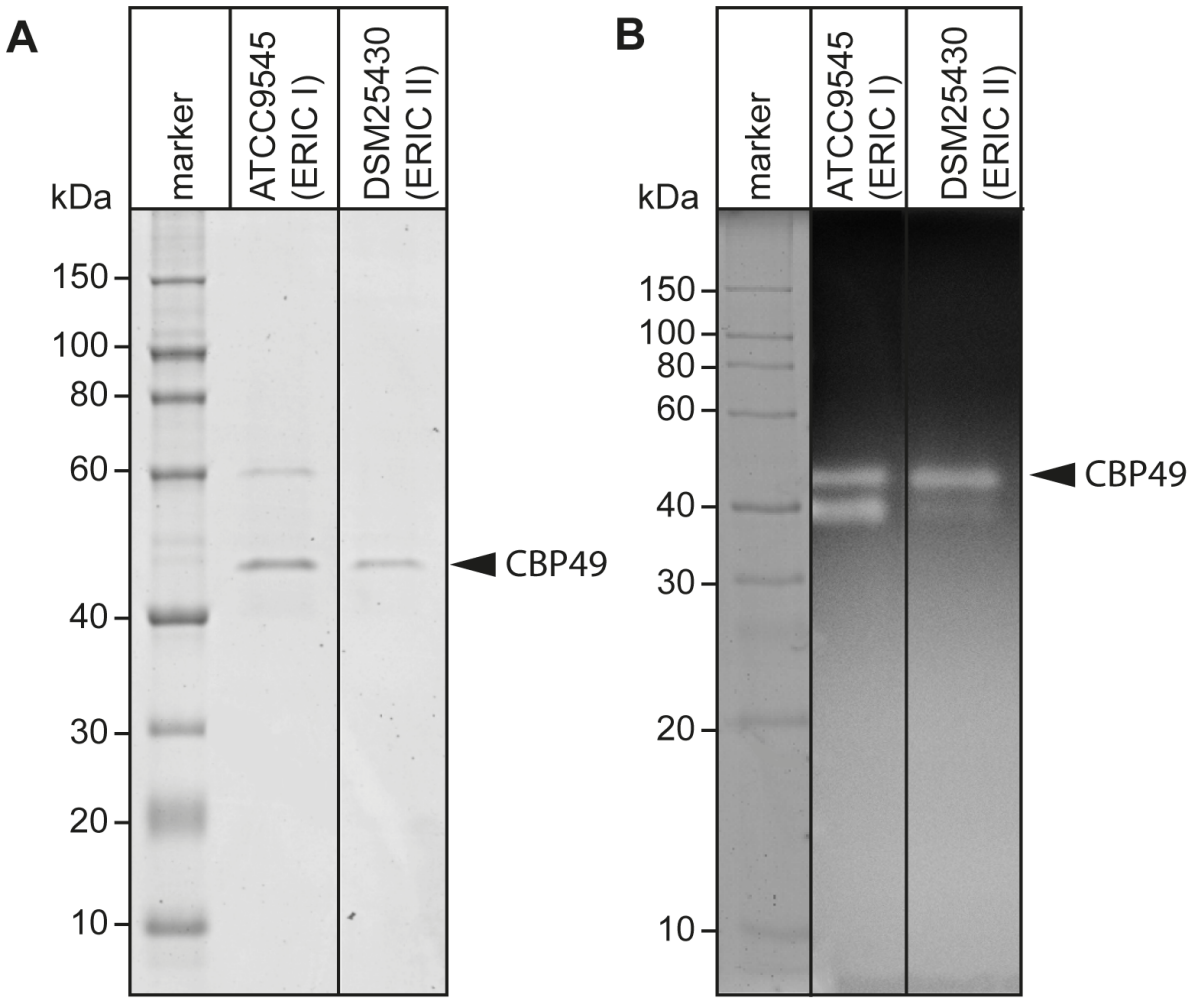
Identification of chitin-binding and –degrading proteins in the supernatants of cultured *P. larvae*. (A) Chitin-binding proteins isolated via chitin-beads from culture supernatants of ATCC9545 (*P. larvae* ERIC I) and DSM25430 (*P. larvae* ERIC II) were subjected to SDS-PAGE analysis. *Pl*CBP49 is marked by an arrowhead. (B) Proteins isolated via chitin-beads from culture supernatants of ATCC9545 (*P. larvae* ERIC I) and DSM25430 (*P. larvae* ERIC II) were subjected to zymography using ethylene glycol chitin- (EGC-) impregnated gels to assess their chitinolytic activity. *Pl*CBP49 is marked by an arrow head.

Recently, degradation of the larval midgut PM by *P. larvae* was demonstrated to be a key step in the pathogenesis of *P. larvae* infections [19]. In order to identify proteins which might be involved in this process, we isolated and analyzed chitin-binding proteins from *P. larvae* culture supernatants using chitin-coated beads. SDS-PAGE analysis of the chitin-binding fractions revealed two chitin-binding proteins (CBP) migrating around 60 kD (CBP60) and 49 kD (*Pl*CBP49_I_) secreted by ATCC9545 (*P. larvae* ERIC I), while only one band migrating around 49 kD (*Pl*CBP49_II_) was visible in the supernatant of DSM25430 (*P. larvae* ERIC II) (Fig. 1A).

To determine the chitinolytic activity of the isolated proteins, the chitin-binding fractions were subjected to zymography performed with denaturing gels containing ethylene glycol chitin (EGC) as the substrate (Fig. 1B). The chitin binding fractions of both isolates produced a clear zone in the range of 49 kD suggesting that in both strains a protein migrating around 49 kD was able to degrade the chitin analogue EGC. We hypothesized that these bands corresponded to *Pl*CBP49_I_ and *Pl*CBP49_II_ identified in SDS-PAGE analysis of the chitin-binding fractions of ATCC9545 and DSM25430, respectively. In addition, chitinolytic activity could also be detected around 42 kD in the chitin-binding fraction of ATCC9545 and a similar activity was weakly but not reproducibly detectable in the chitin-binding fraction of DSM25430. These activities could not be related to any proteins detectable in the Coomassie-stained gels of the chitin-binding fractions hampering their further characterization. This phenomenon was not surprising because zymography is a highly sensitive method of detecting enzymatically active proteins even when present in such low amounts that they cannot be visualized with common staining procedures. In the chitin-binding fraction of ATCC9545, no chitinolytic activity could be observed around 60 kD indicating that CBP60, although binding to chitin, was not able to degrade EGC.

### *Pl*CBP49, the major chitin-degrading enzyme of *P. larvae*, is a member of the AA10 family of LPMOs

**Figure 2:**
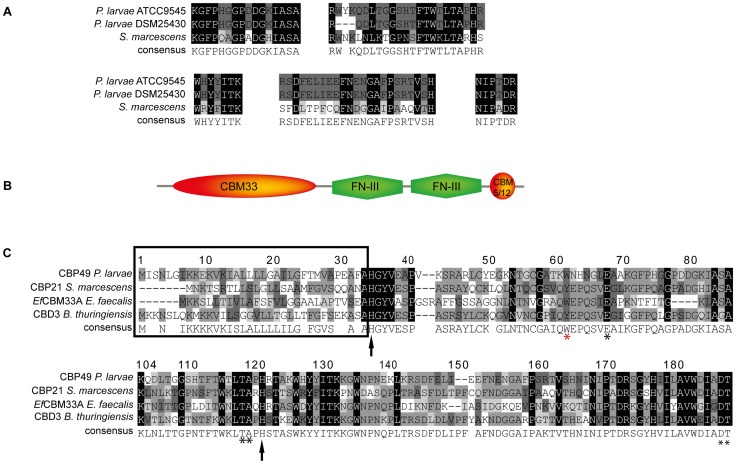
Identification of *Pl*CBP49 as a novel member of the AA10 (formerly CBM33) family of LPMOs. (A) Peptide sequences obtained from sequencing *Pl*CBP49_I_ from ATCC9545 and *Pl*CBP49_II_ from DSM25430 are shown in comparison to the corresponding sequences of *S. marcescens* CBP21 (GenBank acc. no.: BAA31569). (B) *In silico*-translation of the putative *P. larvae Pl*CBP49 ORF followed by domain analysis revealed the presence of an N-terminal CBM33 (AA10) module, two FN-III-repeats and an additional small, C-terminal chitin-binding domain (CBM 5/12). (C) Amino acid alignment of the AA10 domain of *P. larvae Pl*CBP49 with three other members of the AA10 family of LPMOs (CBP21, GenBank acc. no.: BAA31569; *Ef*CBM33A, GenBank acc. no.: AAO80225; CBD3, Genbank acc. no.: EEM95937) revealed the existence of a signal peptide (framed) and several conserved amino acids (arrows and asterisks) which are described to be involved in chitin-binding and –degradation.

To further substantiate that *Pl*CBP49_I_ and *Pl*CBP49_II_ are not only chitin-binding but also chitin-degrading proteins, we determined their protein sequences via mass spectrometry analysis after separation of the bead-bound proteins by SDS-PAGE. Comparison of the obtained peptide sequence data suggested that *Pl*CBP49_I_ was identical to *Pl*CBP49_II_ (Fig. 2A). Protein BLAST analysis of the peptide sequences indicated that *Pl*CBP49_I_ and *Pl*CBP49_II_ were not classical chitinases but rather homologs of CBP21, a chitin-binding and –degrading protein of the chitinolytic bacterium *S. marcescens* belonging to the AA10 family of LPMOs [5], [8], [10] (Fig. 2A).

To analyze whether ATCC9545 and DSM25430 harbor a common gene putatively coding for *Pl*CBP49 we determined the sequences of the corresponding genes *cbp49*_I_ and *cbp*49_II_ in ATCC9545 and DSM25430, respectively, by comparing the obtained peptide fragment sequences with the genomic sequences of *P. larvae* BRL 230010 [27] via TBLASTN analysis [28]. Nucleotide sequence analysis confirmed the presence of the same functional open reading frame (ORF) in both strains (Genbank accession numbers JX185746 for ATCC9545, JX185745 for DSM25430) indicating that both strains harbor the gene *cbp*49 coding for the protein *Pl*CBP49 with 443 amino acids. Screening a collection of *P. larvae* field isolates for the presence of *cbp*49 confirmed the presence of this gene in all strains analyzed so far (data not shown).

Protein BLAST alignment of the translated genomic sequence of *Pl*CBP49 followed by domain analysis of the deduced amino acid sequence using the Conserved Domain Database (CDD; NCBI) revealed a modular protein with an N-terminal domain homologous to AA10 (formerly CBM33) family members of LPMOs followed by two fibronectin type III-like domains, which are often found in bacterial glycosyl hydrolases, and a second small chitin-binding domain (CBM 5/12) at the C-terminus, which is also found in many different glycosyl hydrolase enzymes and presumed to have a carbohydrate binding function (Fig. 2B). Similar domain architecture has recently been described for several other members of the AA10 family of LPMOs [29]–[31]. Amino acid alignment of the AA10 domain of *P. larvae Pl*CBP49 protein with other AA10 family members (Fig. 2C) identified an N-terminal signal sequence (framed) and two histidine residues (His35, His122; arrows) which are highly conserved in all proteins of the AA10 family and which are involved in coordinating copper [8], [10]. Some other conserved residues implicated in binding to chitin [5] were also present such as Tyr62, Glu68, Thr119, Ala120, Asp188, and Thr189 (asterisks) [8], [10] with Tyr62 in *P. larvae* (red asterisk) corresponding to Trp56 in CBP21 and Tyr54 in *Ef*CBM33A [8], [10]. These results indicated that *P. larvae Pl*CBP49 contains a domain characteristic for the AA10 family of LPMOs and suggested that *Pl*CBP49 has LPMO activity.

### *Pl*CBP49 degrades chitin via a metal ion-dependent, oxidative mechanism

**Figure 3:**
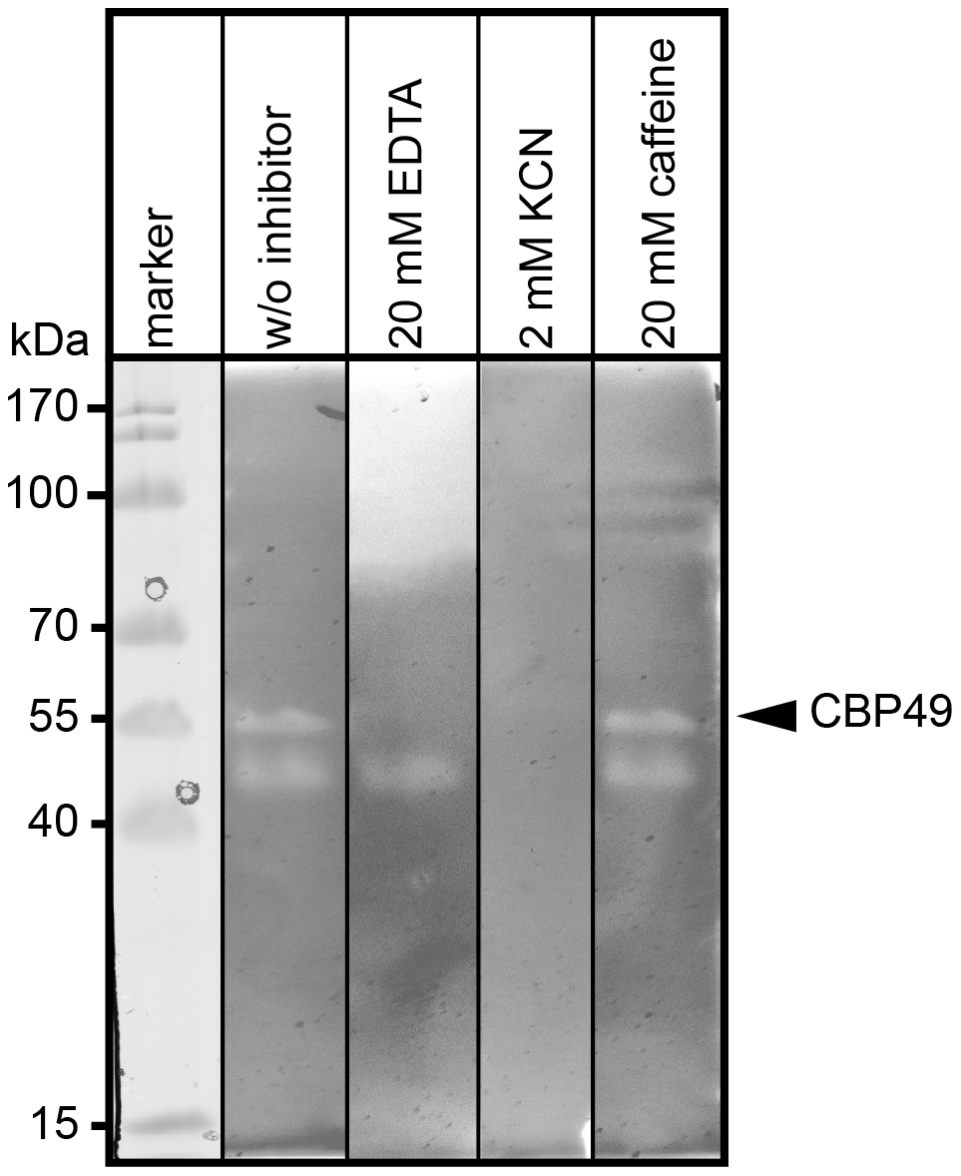
Inhibition of *Pl*CBP49 chitin degradation through KCN and EDTA but not through caffeine. Chitin-binding fractions of *P. larvae* were subjected to zymography and activity of *Pl*CBP49 was probed by adding 20 mM EDTA or 2 mM KCN or 20 mM caffeine as inhibitors. CBP49 activity could be inhibited by EDTA and KCN but not by caffeine. A representative result obtained with the chitin-binding fraction of ATCC9545 (*P. larvae* ERIC I) is shown.

To further substantiate that *P. larvae Pl*CBP49 is a member of the AA10 family of LPMOs we analyzed the chitin-degrading activity of *Pl*CBP49 in greater detail by zymography in the presence of the metal chelator ethylenediaminetetraacetic acid (EDTA) and the di-oxygen mimic cyanide, which were both shown to be potential inhibitors of AA10 family members [8], [9]. *P. larvae Pl*CBP49 activity was strongly inhibited in the presence of 20 mM EDTA and 2 mM potassium cyanide (KCN) (Fig. 3) supporting the involvement of divalent cations and the crucial role of the oxidative step in chitin degradation through *Pl*CBP49. In contrast, presence of 20 mM caffeine, a competitive inhibitor of chitinases [32], did not inhibit chitin degradation (Fig. 3) suggesting that *Pl*CBP49 indeed is not a classical chitinase.

### *Cbp*49 gene disruption results in loss of both *Pl*CBP49 expression and chitinolytic activity

**Figure 4:**
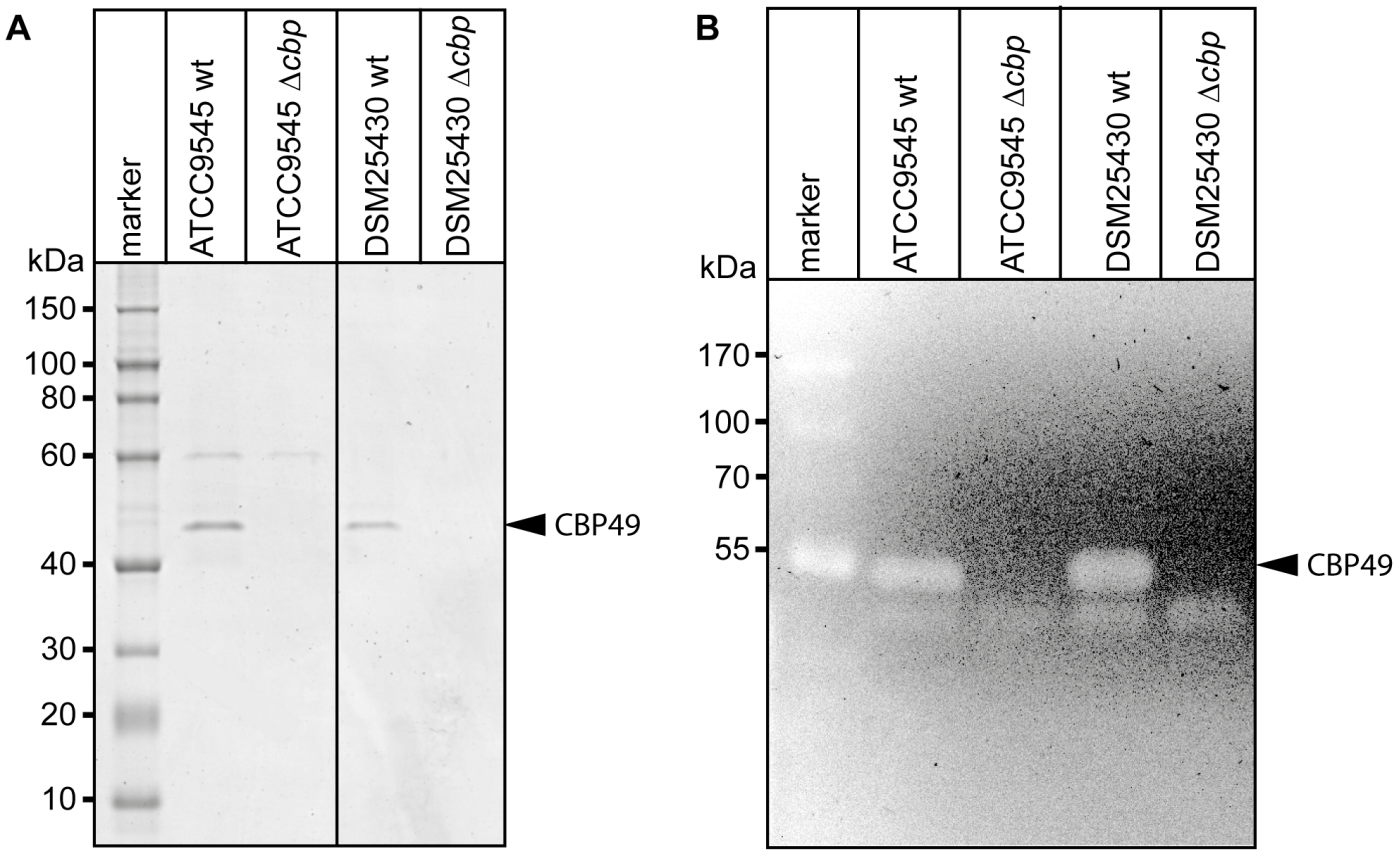
Interruption of the *cbp*-gene in ATCC9545 and DSM25430 leads to lack of both *Pl*CBP49 expression and chitinolytic activity. (A) SDS-PAGE analysis of the culture supernatant fractions bound to chitin coated beads of wild-type (ATCC9545, DSM25430) and mutant bacteria (ATCC9545 Δ*cbp*, DSM25430 Δ*cbp*) confirmed the absence of *Pl*CBP49 expression in the knockout strains. Bands corresponding to *Pl*CBP49 are marked by an arrow head. (B) Zymography of the culture supernatants of wild-type (ATCC9545, DSM25430) and mutant bacteria (ATCC9545 Δ*cbp*, DSM25430 Δ*cbp*) confirmed the absence of *Pl*CBP49 activity in the knockout strains. Bands corresponding to *Pl*CBP49 chitinolytic activity are marked by an arrow head.

To better analyze the chitin-degrading activity of *Pl*CBP49, to functionally characterize this protein in the *P. larvae* genotypes ERIC I and ERIC II [13], [16], and to assess its role in the pathogenesis of AFB, *P. larvae* mutants deficient in the expression of *Pl*CBP49 were constructed from ATCC9545 and DSM25430 as parent strains. The chitin-binding fractions of both mutant strains were analyzed by SDS-PAGE (Fig. 4A) and zymography (Fig. 4B) to demonstrate loss of *Pl*CBP49 expression and of chitinolytic activity concomitant with *cbp*49 gene disruption. In both mutants, ATCC9545 Δ*cbp* and DSM25430 Δ*cbp*, the protein bands corresponding to *Pl*CBP49 (Fig. 4A) as well as the chitinolytic activity migrating around 49 kDa (Fig. 4B) were missing in the corresponding chitin-binding fractions. In contrast, the chitinolytic activity visible around 42 kDa was unaffected (Fig. 4B) indicating that gene disruption of *cbp*49 had no downstream effects on the chitinolytic machinery detectable via zymography. These results clearly confirmed that the identified *cbp*49 gene encodes the identified protein *Pl*CBP49 which in turn is responsible for the observed activity towards soluble chitin.

### *P. larvae Pl*CBP49 is involved in PM degradation

**Figure 5:**
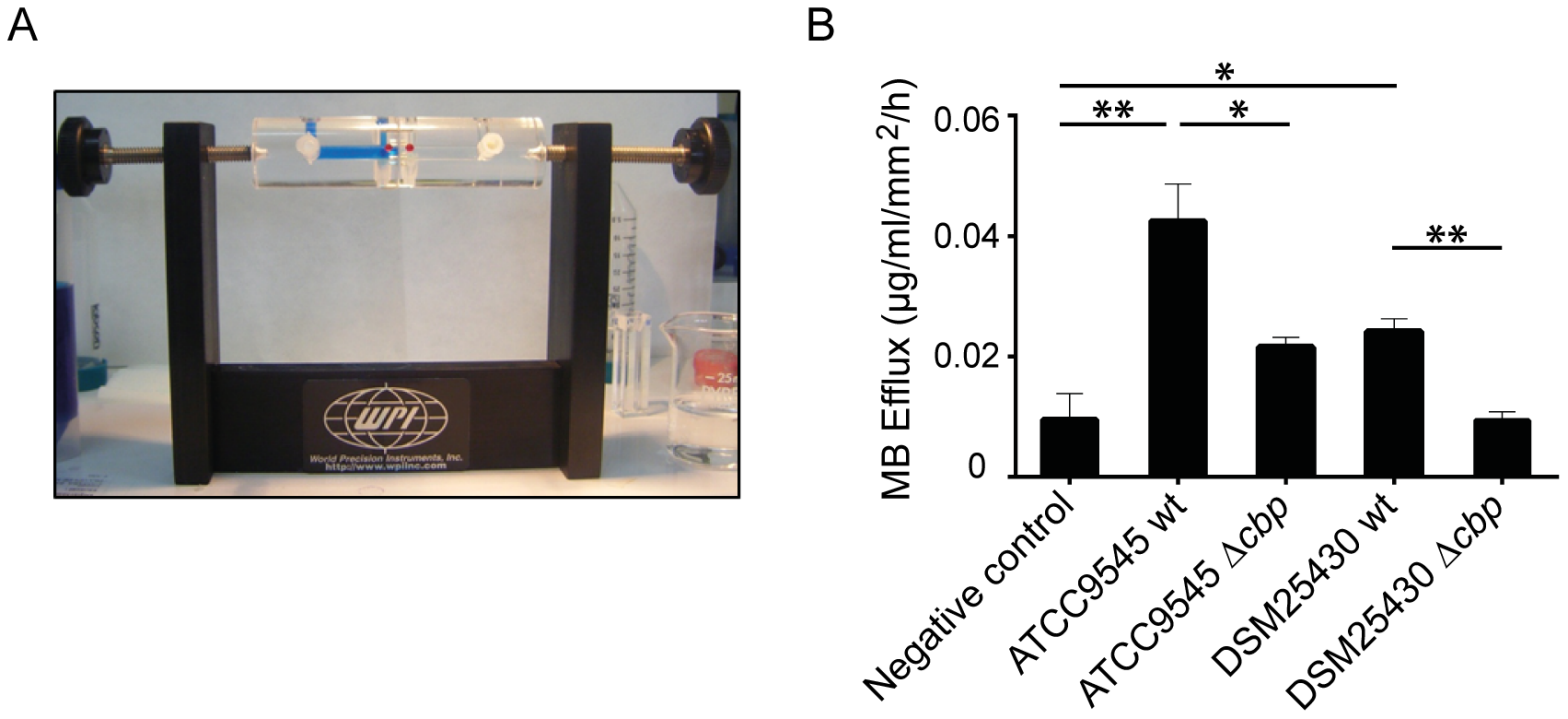
Degradation of insoluble chitin structures by *P. larvae Pl*CBP49. Peritrophic matrices were isolated from *Spodoptera frugiperda* larvae and incubated in an Ussing-chamber (A) with mock-treated chitin beads as negative control and chitin-binding fractions of either wild type bacteria or the corresponding mutant bacteria. Methylene blue efflux was used as a measure for permeability (B). PM permeability was significantly higher than negative control after incubation with ATCC9545 wt and DSM25430 wt chitin binding fractions. After incubation with ATCC9545 Δ*cbp* and DSM25430 Δ*cbp* chitin-binding fractions PM permeability was significantly lower compared to the incubation with chitin-binding fractions of the wild-type strains (ATCC9545, DSM25430). Bars represent mean values + SEM of at least three independent experiments, analyzed by student's t-test; *p value<0.05 and **p value<0.01.

We recently showed that *P. larvae* is able to metabolize insoluble, colloidal chitin [19]. However, the data related to the chitin-degrading activity of *Pl*CBP49 obtained so far were based on using EGC as a soluble substrate in zymograms. To further verify that degradation of insoluble recalcitrant polysaccharides is mediated by *Pl*CBP49, as it is described for members of the AA10 family of LPMOs, we tested whether or not *Pl*CBP49 might be able to degrade chitin-containing structures like an insect PM. To this aim, we used an Ussing chamber (Fig. 5A) to perform permeability assays with PMs which were isolated from *S. frugiperda* last instar larvae and subjected to the chitin bound fractions of the knock-out *P. larvae* strains (ATCC9545 Δ*cbp*, DSM25430 Δ*cbp*) and of the corresponding parent wild-type strains (ATCC9545, DSM25430). This comparative approach allows differences in PM permeabilization between mutant and wild-type bacteria to be linked with differences in *Pl*CBP49 expression. Permeability of the PMs was measured as methylene blue (MB) efflux and was significantly higher after incubation with the chitin-binding fractions of the wild-type bacteria than after incubation with the chitin-binding fractions of the corresponding mutants (Fig. 5B). For *P. larvae* ATCC9545 MB efflux significantly (student's t-test, p-value = 0.0125) decreased from 0.0429±0.006 µg/ml/mm^2^/h in the presence of *Pl*CBP49 expression (ATCC9545, mean values ± SEM) to 0.021±0.0016 µg/ml/mm^2^/h in the absence of *Pl*CBP49 expression (ATCC9545 Δ*cbp*, mean values ± SEM). Similar results were obtained for *P. larvae* DSM25430 (0.02418±0.002 µg/ml/mm^2^/h, mean values ± SEM) compared to *P. larvae* DSM25430 Δ*cbp* (0.0094±0.0013 µg/ml/mm^2^/h, mean values ± SEM) which were also significantly different (student's t-test, p-value = 0.004). Remarkably, exposure of PMs to chitin-bound fractions of DSM25430 Δ*cbp* resulted in PM permeability not significantly different from the negative control (student's t-test, p-value = 0.983) indicating that in the absence of *Pl*CBP49 expression no PM degrading activity was active in these fractions. In contrast, chitin-bound fractions of ATCC9545 Δ*cbp* resulted in PM permeability that was significantly higher than the negative control (student's t-test, p-value = 0.006) although also significantly reduced when compared to the effect achieved with ATCC9545 wild-type, meaning in the presence of *Pl*CBP49 expression. These results indicated that the chitinolytic activity of *Pl*CBP49 can act on chitin in its native crystalline form suggesting a role for *Pl*CBP49 also in PM degradation observed during *P. larvae* infection of honey bee larvae [19].

### *Pl*CBP49 is an essential virulence factor of *P. larvae*

**Figure 6:**
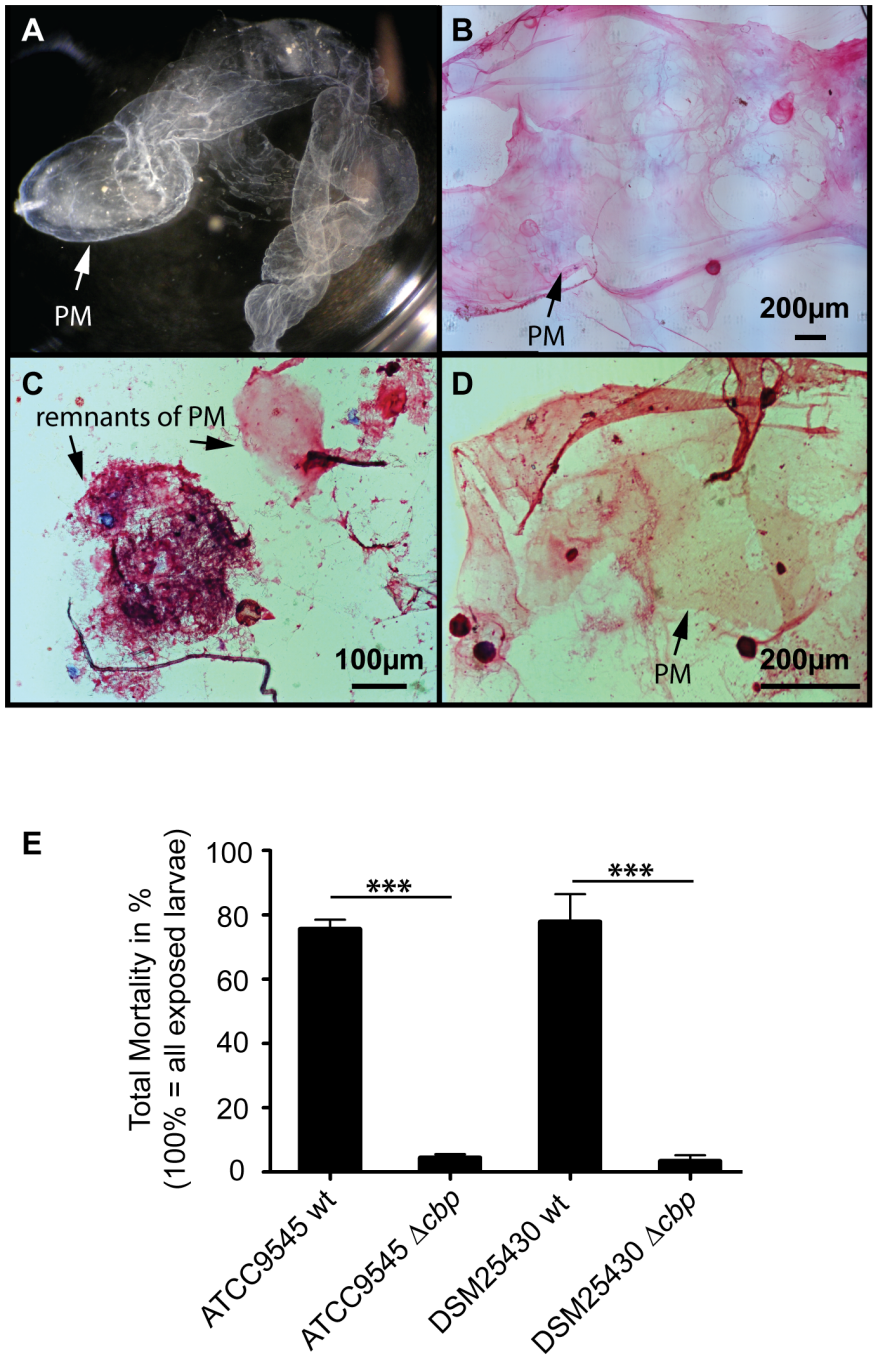
Knocking out *PlCBP49* expression impairs PM degradation in infected larvae and nearly abolishes *P. larvae* virulence. (A) PM from 6-day old non-infected control larvae can be isolated as nearly intact structures. A representative macroscopic picture of an unstained PM is shown. (B) After methylene blue-basic fuchsine staining of chitin, the isolated PM of non-infected control larvae could be visualized as intact, pink stained structure. (C) Methylene blue-basic fuchsine staining of PM isolated from 6-day old larvae infected with ATCC9545 wild type bacteria revealed no intact structure but instead only red stained dots most likely remnants of the degraded PM can be observed. (D) Methylene blue-basic fuchsine staining of PM isolated from 6-day old larvae infected with the mutant strain ATCC9545 Δ*cbp* showed an intact, pink stained PM structure. (E) Larvae were infected with wild type bacteria (ATCC9545, DSM25430) or the corresponding mutant strains (ATCC9545 Δ*cbp*, DSM25430 Δ*cbp*) and total mortality was calculated for each group. Data represent mean values + SEM of three independent infection assays with 30 larvae each. Data were analyzed by student's t-test; ***p<0.001.

To test the proposed involvement of *Pl*CBP49 in PM degradation in *P. larvae* infected honey bee larvae, we isolated (Fig. 6A) and stained PMs (Fig. 6B–D) from non-infected control larvae as well as from larvae infected with either wild-type *P. larvae* (ATCC9545) or mutant *P. larvae* (ATCC9545 Δ*cbp*) in order to assess PM integrity. While control larvae at the age of six days after egg hatching contained intact PMs (Figs. 6A, B), the PM in ATCC9545-infected larvae of the same age appeared almost totally degraded with only small patches of stainable structures (Fig. 6C). In contrast, larvae of the same age infected with ATCC9545 Δ*cbp* and, therefore, lacking the chitin-degrading activity of *Pl*CBP49, did still contain an almost intact and stainable PM (Fig. 6D). This result indicated that PM degradation only occurs in the presence of *Pl*CBP49 expression and, therefore, confirmed that *Pl*CBP49 plays a key role in the degradation of the larval PM during infection.

PM degradation in *P. larvae* infected larvae has been shown to be a key step in AFB pathogenesis [19]. Therefore, we hypothesized that *Pl*CBP49 involved in PM degradation in infected larvae should be a key virulence factor of this pathogen. To test this, we performed laboratory infection assays by feeding first instar honey bee larvae larval diet containing spores of the knock-out *P. larvae* strains (ATCC9545 Δ*cbp*, DSM25430 Δ*cbp*) and of the corresponding parent wild-type strains (ATCC9545, DSM25430). Comparing mortality in the groups infected with the wild-type and with the mutant bacteria revealed that the lack of *Pl*CBP49-expression led to a significant reduction in mortality (Fig. 6E). While 500 cfu/ml of ATCC9545 resulted in a total mortality of 75.5%±5.0% (mean values ± SEM), the same spore concentration killed only 4.4%±1.1% (mean values ± SEM) in the group infected with the mutant strain ATCC9545 Δ*cbp* (student's t-test, p-value = 0.0001). The results were similar for DSM25430 and DSM25430 Δ*cbp*: 100 cfu/ml killed 77.7%±8.7% (mean values ± SEM) in the group infected with the wild-type DSM25430 and only 3.3%±1.9% (mean values ± SEM) died in the group infected with DSM25430 Δ*cbp* (student's t-test, p-value = 0.0011). In other words, lack of *Pl*CBP49 activity resulted in about 94% reduction in mortality for ATCC9545 and nearly 96% reduction in mortality for DSM25430. Therefore, the lack of *Pl*CBP49 expression nearly abolished the virulence of both *P. larvae* strains. These results clearly indicated that *Pl*CBP49 is a key virulence factor for both *P. larvae* genotypes.

## Discussion

Attempts to understand the pathogenesis of *P. larvae* infections of honey bee larvae have been undertaken since the early days of AFB research. It was suggested that the PM acts as a protective shield against *P. larvae* infections because it might hamper vegetative bacteria in their attempts to cross the gut wall [17], [33]–[35]. Recently, we demonstrated that the PM indeed is the first barrier vegetative *P. larvae* bacteria have to overcome before attacking and breaching the epithelium [20]. In addition, we recently showed that *P. larvae* is able to metabolize colloidal chitin [19] and that the larval midgut PM contains chitin which is degraded during *P. larvae* infection, leading to loss of PM integrity [19]. We now describe the identification and functional analysis of *P. larvae Pl*CBP49, a new member of the AA10 (formerly CBM33) family of chitinolytic LPMOs which were recently shown to be able to degrade crystalline chitin via a novel, oxidative mechanism [8]. We demonstrate that *Pl*CBP49 is involved in PM degradation in infected honey bee larvae and that it is a key virulence factor for both *P. larvae* genotypes, as in the absence of *Pl*CBP49 expression *P. larvae* virulence was almost lost (for a definition of the term virulence please see [36]).

When invading the infected larva, *P. larvae* faces two physical barriers: first the peritrophic membrane and second the epithelium [20]. Although the destruction of the epithelial cell layer by *P. larvae* is still poorly understood, the identification and characterization of *Pl*CBP49 now allows the biochemical mechanism by which *P. larvae* penetrates and traverses the PM to be unravelled. *Cbp*49 encodes a chitin-binding and –degrading protein belonging to the AA10 (formerly CBM33) family of chitin-degrading LPMOs. The best analyzed member of this family is CBP21 expressed by *S. marcescens*, a Gram-negative soil bacterium described as an insect pathogen but which is also an opportunistic pathogen of mammals [6], [37], [38]. CBP21 was originally described as lacking any catalytic activity of its own despite its essential role in chitin-degradation [5]. However, it was recently shown to possess chitin-degrading activity [8] and it was demonstrated that CBP21 degrades crystalline chitin through a novel copper-dependent oxidative mechanisms [8], [10].

So far, the only function attributed to CBP21 is that of a food-scavenging enzyme allowing *S. marcescens* to use chitin as carbon source. However, *S. marcescens* is pathogenic to many invertebrates including insects where it causes intestinal infections [37]. Based on our results we now propose that the biological role of *S. marcescens* CBP21 is not only to enable these bacteria to use chitin as carbon source but also to allow them to degrade the protective PM as part of the pathogenic process when *S. marcescens* acts as insect pathogen. This assumption is further substantiated by recent findings showing that *Drosophila melanogaster* deficient in forming a proper PM due to a loss-of-function mutation in the gene *dcy* coding for Drosocrystallin, an integral component of *D. melanogaster* PM, are more susceptible to oral infection by *S. marcescens* than wild-type flies [39]. Furthermore, for *Ef*CBM33A it was recently speculated that it is involved in host-microbe interactions based on the observed gene regulation [9].

*Pl*CBP49 is 443 amino acids in length and in addition to the AA10 module also contained two fibronectin type III-like domains and another chitin-binding module, CBM5/12. The latter domain has often been found in proteins expressed by bacteria thriving in the digestive tract of invertebrates [40]. For instance, CBP50 expressed by *B. thuringiensis* has a similar domain architecture [30] and it was demonstrated that the FN III-like domains and the CBM5/12 domain are involved in substrate binding. Future experiments should address the question of the function of these domains for *Pl*CBP49 activity. According to our results, *Pl*CBP49 not only degraded insoluble chitin structures but also acted on EGC, a soluble analog of chitin. Further biochemical and molecular studies are needed to unravel the function of these additional modules in the context of LPMO activity and to analyze whether these modules are involved in cleavage of soluble chitin, a capability not yet described for other members of the AA10 family of LPMOs.

Normally, *Bacilli* and *Paenibacilli* express a wide range of classical chitinases. However, recent evidence suggests that the situation is totally different for *P. larvae*. When the entire genomes of two *P. larvae* strains representing the genotypes ERIC I (DSM25719) and ERIC II (DSM25430) were sequenced, manually curated, annotated, and searched for putative virulence factors, in both genomes only a pseudogene containing a chitinase A (GH18 family) N-terminal domain was identified. While the *P. larvae* genomes harbored more than 100 protease genes, genes coding for classical chitinases were missing [26]. These most recent *in silico* results further support our finding that *Pl*CBP49 is a key chitin-degrading protein of *P. larvae*.

By using a chitin-binding activity based approach, we identified *Pl*CBP49 as chitin-degrading protein and we demonstrated that *Pl*CBP49 is able to degrade both soluble and insoluble chitin and is essential for PM degradation *in vitro* and *in vivo*. However, while in the Ussing chamber experiment gene disruption of *cbp*49 in DSM25430 resulted in loss of *S. frugiperda* PM degradation by DSM25430 Δ*cbp*, the chitin bound fraction of ATCC9545 Δ*cbp* still seemed to contain some activity affecting PM integrity. We speculate that this activity might be due to CBP60, present only in ATCC9545 and ATCC9545 Δ*cbp* chitin bound fractions but absent in the corresponding DSM25430 fractions (Fig. 1). Although CBP60 is a chitin-binding protein, it did not show any chitin-degrading activity. However, CBP60 might have protease activity targeting non-chitin components of the PM like insect intestinal mucin (IIM) or other proteins thereby affecting PM integrity. More than hundred putatively functional protease genes were present in the *P. larvae* genomes [26] leaving abundant CBP60-candidates which might contribute to PM degradation in ATCC9545. Obviously, much further work is needed to unravel and understand the entire PM degrading system of ATCC9545, to identify CBP60, and to analyze whether it plays a role in PM degradation. Identification and characterization of *Pl*CBP49 presented here is a first and important step towards this end.

Unfortunately, it was impossible to construct complementation mutants to provide the final control for the assays performed with mutant *P. larvae*. However, our data (i) on PM degradation during *P. larvae* infection [19], (ii) on the lack of classical chitinase genes in the genome of *P. larvae* [26], and (iii) the combination of *in vitro* and *in vivo* data presented here provide a convincing body of evidence for our interpretation of the role and relevance of *Pl*CBP49 in AFB pathogenesis.

Weakening or even destroying the larval PM allows more ready access of bacteria or bacterial toxins to gut epithelial cells [3], [39], [41]. We recently described the identification of several putative toxin genes in the genome of *P. larvae* ERIC I [42] and the detailed analysis of two of these toxins, Plx1 and Plx2, which belong to the family of AB-toxins [43]–[45]. Binding of *P. larvae* AB-toxins to their cognate cell surface receptors of the midgut epithelial cells might be accomplished more easily or even only when the epithelium is no longer protected by a PM like already shown in other insect systems [3]. We, therefore, suggest that degradation of the PM is a prerequisite for *P. larvae* AB-toxins Plx1 and Plx2 to act on the epithelial cells.

We recently identified SplA, a *P. larvae* ERIC II-specific surface-layer (S-layer) protein which presumably mediates bacterial adhesion to midgut epithelial cells [46]. Direct adhesion of *P. larvae* ERIC II to host cells via SplA might only be possible if the cells are no longer protected by a PM. We, therefore, suggest that in the case of *P. larvae* ERIC II, degradation of the PM is a prerequisite for the bacteria to directly approach the epithelial layer.

Based on these scenarios we propose the following model for pathogenesis of *P. larvae* infections: during the initial, non-invasive phase of infection, degradation of the PM serves nutritional needs and enables *P. larvae* to use chitin as an additional carbon source. However, at some time point the PM is totally degraded and bacteria (e.g. via SplA for *P. larvae* ERIC II) and/or bacterial toxins (Plx1 and Plx2 for *P. larvae* ERIC I) can directly approach and act on the host cells leading to bacterial breaching of the epithelium and initiation of the invasive phase. According to this model, blocking PM degradation blocks the transition from the non-invasive phase to the invasive phase explaining the impressive loss of virulence connected with the inactivation of *Pl*CBP49 activity. This makes *Pl*CBP49 a key virulence factor of *P. larvae*.

## Materials and Methods

### Bacterial strains and growth conditions

*Paenibacillus larvae* strains ATCC9545 and DSM25430 representing the two *P. larvae* genotypes ERIC I and ERIC II [13], respectively, were used in this study. Strain ATCC9545 is the type reference strain and was obtained from the American Type Culture Collection (ATCC, USA) through U. Rdest (Biocenter Würzburg, Germany). Strain DSM25430 (Deutsche Sammlung von Mikroorganismen) corresponds to the field isolate 04-309 originating from an outbreak of American Foulbrood in Germany [47]. Both strains have been characterized in several previous studies [13], [42], [46], [48]–[50]. Non-manipulated *P. larvae* wild-type bacteria were cultivated either in MYPGP liquid broth or on Columbia sheep blood agar plates at 37°C as previously described [47], [51]. Manipulated knockout clones were cultivated on MYPGP-agar plates [52] supplemented with 5 µg/ml chloramphenicol and incubated at 37°C for 2–3 d as previously described [46].

*Escherichia coli* DH5α cells (Invitrogen) transformed with plasmids pTT_*wsf*A243 [53] or pTT_*cbp*573 (see below) were cultivated in selective Luria Bertani (LB) media (agar and broth) supplemented with 30 µg/ml chloramphenicol. Plasmid DNA was prepared following the manufacturer's protocols (QIAprep Spin Miniprep kit, Qiagen). Concentration and purity of the plasmid preparations were analyzed by photometric analysis (Nanodrop) and agarose gelelectrophoresis.

Preparation of spore suspensions for exposure bioassays and determination of spore concentrations by cultivating serial dilutions on Columbia sheep blood agar plates were performed as described previously [13], [48], [49].

### Protein purification and analysis

For small-scale affinity purification of *P. larvae* proteins exhibiting a chitin-binding domain (CBD), *P. larvae* was grown under the conditions described above and the culture supernatant was collected in the stationary phase by centrifugation. Aliquots (50 µl) of magnetic beads coated with chitin (chitin magnetic beads, New England Biolabs) were washed twice with CBD Column Binding Buffer (500 mM NaCl, 20 mM Tris-HCl, 1 mM EDTA, 0.05% Triton X-100; pH 8.0) at 25°C. An aliquot of filtered (2 µm-filter, Roth) *P. larvae* culture supernatant (500 µl) was incubated with an aliquot of pre-washed beads for 1 h at 4°C with mild agitation. Beads were magnetically collected and subsequently washed three times with CBD buffer, resuspended in non-reducing sodium dodecyl sulfate–polyacrylamide gel electrophoresis (SDS-PAGE) sample buffer [54], heated for 5 min at 96°C to release the chitin-binding proteins which were then separated by SDS–PAGE and analyzed after staining with Coomassie Brilliant Blue.

Chitin-degrading activity was analyzed by zymography in 10% SDS-PA gels containing 0.1% ethylene glycol chitin (EGC) according to Trudel and Asselin [55]. Briefly, chitin-bound fractions were mixed with non-reducing SDS-PAGE sample buffer [54], heated for 5 min at 96°C to release the chitin-bound proteins and subjected to SDS-PAGE. Subsequently, gels were incubated with mild agitation at 37°C for 2 h in sodium acetate buffer (NaAc buffer; pH 5) containing 1%Triton X-100. Gels were stained for 5 min with 0.01% calcofluor in 0.5 M Tris-HCl (pH 9) and washed four times for 15 min with deionised water. Gel analysis was performed under UV-light. For analyzing the reaction mechanism of *Pl*CBP49, zymography was also performed with NaAc buffer supplemented with 2 mM potassium cyanide (KCN), 20 mM ethylenediaminetetraacetic acid (EDTA), or 20 mM caffeine to determine the conditions inhibiting chitin-degrading activity of *Pl*CBP49.

### Determination of the protein and nucleic acid sequence of *Pl*CBP49

In order to determine the protein sequence of *Pl*CBP49_I_ and *Pl*CBP49_II_, chitin-binding fractions of ATCC9545 and DSM25430 were separated via SDS-PAGE. Coomassie stained bands of both, *Pl*CBP49_I_ and *Pl*CBP49_II_ were excised from the gel and analyzed by mass spectrometry (Alphalyse, Denmark) as already described [50]. The provided protein sequence analysis service included reduction and alkylation of cysteine residues, digestion of the proteins with trypsin, extraction and micro-purification of the obtained peptides using C18 ziptips followed by peptide mapping via MALDI-ToF and partial peptide sequencing via MALDI-ToF/ToF. Proteins were identified on the basis of peptide masses and sequence information by using the in-house databases of Alphalyse (Denmark) and the NCBI database. The obtained peptide sequences of *Pl*CBP49_I_ and *Pl*CBP49_II_ were compared to each other using the protein alignment tool of Vector NTI (Invitrogen).

To identify the genetic information belonging to the sequenced peptides in *P. larvae*, obtained peptide fragment sequences were compared by TBLASTN analysis [28] with the sequence of *P. larvae* BRL 230010 [27]. The best hit was with the sequence ZP_09067740.1 annotated as chitin-binding protein. The corresponding ORF was highly homologous to a hypothetical protein of *Bacillus cereus* (EJQ09528.1; E-value 4e-161) and chitin-binding domain 3 protein of *Bacillus thuringiensis* serovar *berliner* (ZP_04102491.1; E-value 4e-161). In order to determine the genomic sequence of *Pl*CBP49 in *P. larvae* ATCC9545 and *P. larvae* DSM25430, we selected a primer pair amplifying the complete predicted ORF of *Pl*CBP49 (cbp_F and cbp_R, Table 1) and a second primer pair located up and downstream of the predicted ORF (cbp_up and cbp_down, Table 1). The obtained amplicons for both genotypes were sequenced (Eurofins, Germany) and sequences were aligned using the DNA alignment tool of Vector NTI (Invitrogen). PCR-analysis with primer pair cbp_F and cbp_R (Table 1) of several field isolates of *P. larvae* ERIC I and ERIC II confirmed the presence of the *Pl*CBP49-gene in all strains analyzed so far (data not shown).

**Table 1:** Primers used for sequence analysis of *cbp*49, screening of *P. larvae* isolates for *cbp*49, and construction of gene knockouts in *P. larvae* ATCC9545 (ERIC I) and DSM25430 (ERIC II).

primer name	primer sequences	purpose (size of amplicon)
cbp_up	5′- CCGTAGCAGAATTAAAATGAAAGGA -3′	sequencing
cbp_down	5′- TCACTGGGCAGCCGAAATAA -3′	(1537 bp)
cbp_F	5′- ATGATCTCTAATTTGGGTATTAAAA -3′	sequencing and screening
cbp_R	5′- TTATTCAATCACCGTCCATA -3′	(1344 bp)
IBS_cbp_573	5′- AAAAAAGCTTATAATTATCCTTAGATACCGGGGCCGTGCGCCCAGATAGGGTG -3′	*cbp* knockout
EBS1d_cbp_573	5-′ CAGATTGTACAAATGTGGTGATAACAGATAAGTCGGGGCCGCTAACTTACCTTTCTTTGT -3′	
EBS2_cbp_573	5′- TGAACGCAAGTTTCTAATTTCGATTGTATCTCGATAGAGGAAAGTGTCT -3′	

### Disruption of the gene coding for *Pl*CBP49 in *P. larvae*

The *Pl*CBP49-gene in the genomes of *P. larvae* ATCC9545 and *P. larvae* DSM25430 was disrupted via a recently described strategy [46], [56] using vector pTT_*wsf*A243 containing the bacterial mobile group II intron LI.LtrB sequence [46], [53] for constructing a targetron vector for targeted intron insertion at position 573/574 from the start codon of *cbp*49 in *P. larvae* determined as optimal insertion site by a computer algorithm provided by the manufacturer (http://www.sigma-genosys.com/targetron). Retargeting of the LI.LtrB targetron of vector pTT_*wsf*A243 prior to transformation into *P. larvae* was performed following the manufacturer's protocol (Sigma) and essentially as already described for disruption of the *P. larvae* S-layer gene *spl*A [46]. In brief, primers IBS_cbp_573, EBS1d_cbp_573, and EBS2_cbp_573 (Table 1) designed by a computer algorithm provided by the manufacturer (see above) were used for modification of the LI.LtrB targetron to generate a specific *cbp*49 targetron. Replacement of the *wsf*A243 targetron in pTT_*wsf*A243 by the *cbp*49 targetron gave rise to the new vector pTT_*cbp*573 which was subsequently transformed into *E. coli* DH5α cells for plasmid replication and preparation.

**Figure 7:**
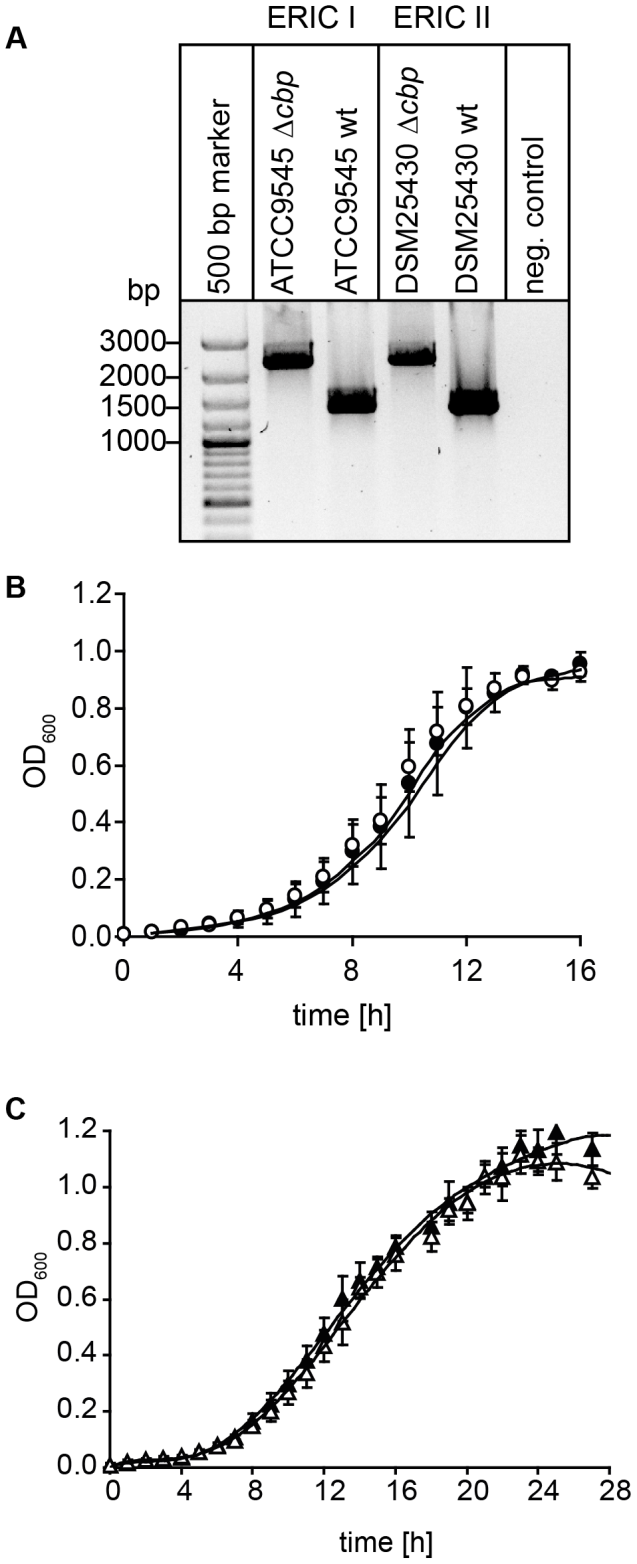
Disruption of the gene coding for *Pl*CBP49 in *P. larvae* ATCC9545 (ERIC I) and in *P. larvae* DSM25430 (ERIC II). (A) Migration properties of amplicons from the wild-type (ATCC9545 wt, DSM25430 wt) and mutant bacteria (ATCC9545 Δ*cbp*, DSM25430 Δ*cbp*) confirmed the successful insertion of the targetron into the gene *cbp*49. (B) Growth curves of the knockout strain ATCC9545 Δ*cbp* (open circle) in comparison to the parent wild-type strain ATCC9545 (closed circle). (C) Growth curves of the knockout strain DSM25430 Δ*cbp* (open triangle) in comparison to the parent wild-type strain DSM25430 (closed triangle). Both results showed that disrupting *Pl*CBP49 expression did not influence bacterial growth.

For creation of *P. larvae* knockout mutants, electrocompetent *P. larvae* ATCC9545 and *P. larvae* DSM25430 cells were prepared as described [57] and 1 µg of plasmid pTT_*cbp*573 was transformed by electroporation as recently established [58]. Positive clones were selected on MYPGP-agar containing 5 µg/ml chloramphenicol. Successful insertion of the *cbp*49-specific retargeted intron (915 bp) into the *P. larvae* target gene *cbp*49 was demonstrated by PCR-analysis of the corresponding genomic regions in both knockout-strains designated *P. larvae* ATCC9545 Δ*cbp* and *P. larvae* DSM25430 Δ*cbp* using primers cbp_F and cbp_R flanking the *cbp*49 intron insertion position 573/574 of the ORF (Table 1) followed by sequencing the obtained PCR products. The mutant amplicons carrying the insertion migrated at 2259 bp whereas the wild-type amplicons had the expected size of 1344 bp (Fig. 7A). Further analyses of the mutant strains in comparison to their respective parent wild type strains were performed as already described [46] and did not reveal any differences in germination, sporulation, and growth in liquid broth (Fig. 7B,C) which otherwise might have influenced functional analyses. Clones *P. larvae* ATCC9545 Δ*cbp* and *P. larvae* DSM25430 Δ*cbp* were further analyzed for the absence of *Pl*CBP49 in the chitin-binding fractions of the secretomes via SDS-PAGE and zymography.

### Isolation of larval peritrophic membrane

Non-infected control larvae as well as larvae infected with wild-type strain ATCC9545 or with *Pl*CBP49 deficient strain ATCC9545 Δ*cbp* were reared in 24-well plates as described below. Larvae at 6 d of age were immobilized for 5 min on ice. Larvae were placed under the binocular and opened longitudinally. Fat body was carefully separated from larval midgut, discarded and midgut was washed with 1XPBS (phosphate buffered saline). Clean midgut was opened lengthways and peritrophic membrane was carefully pulled out. 1 ml of 1XPBS was dispensed per well in a 24-well plate and each PM corresponding to different larvae was disposed in a different well. Afterwards, clean PMs were extended on a microscope slide, dried out and stained with a methylene blue-basic fuchsine staining technique essentially as previously described [19].

### Permeability tests

Changes in PM permeability were measured using an Ussing chamber (CHM8; World Precision Instruments, Stevenage, United Kingdom) and methylene blue (MB) as described before [59]. *Spodoptera frugiperda* was employed as a model insect since the required PM area necessary for performing permeability experiments could not be obtained from honey bee larvae. *S. frugiperda* PMs were isolated from actively feeding last-instar larvae. Each larva was anesthetized shortly on ice; midgut was extracted and longwise opened. PM was carefully pulled out, disposed on a cotton film and opened. The film was cut again to adjust PM containing area and gently washed with 1XPBS to remove food content. Cotton film was then assembled in an Ussing chamber. A suitably sized piece of the PM was used to cover the hole (12.6 mm^2^) in the Ussing chamber separating the two compartments. The ectoperitrophic side of the PM faced the compartment filled up with 400 µl of 0.2 mg/ml methylene blue solution, and the endoperitrophic side faced the second compartment with 400 µl of 1XPBS. Optical density at 661 nm (OD_661_) was measured at the beginning of the experiment and after 30 min to ensure the integrity of the PM. To test the activity of *Pl*CBP49 against the PM, 100 µl of the buffer solution in the endoperitrophic compartment was replaced with an equivalent volume of chitin binding fraction of each *P. larvae* strain (ATCC9545, ATCC9545 Δ*cbp*, DSM25430 and DSM25430 Δ*cbp*). After additional 2 h of incubation the solutions in both compartments were recovered, and the concentration of MB was calculated based on the OD_661_ nm measures. The MB flux was expressed as µg of dye that passed through the 12.6-mm^2^ portion of mounted PM in 1 h. The flux measured at 30 min was subtracted from the final flux, to normalize the initial permeability of the PMs. Negative controls were performed with mock incubated chitin beads to rule out any influence of the beads or the buffers used on the PM. At least three independent replicates were performed for each data collection. Data represent mean values ± SEM. Data were statistically analyzed by student's t-test.

### Exposure bioassays

In order to analyze the functional role of *P. larvae Pl*CBP49 during pathogenesis of *P. larvae* infections, honeybee larvae reared *in vitro* were experimentally infected with the *P. larvae* knockout strains ATCC9545 Δ*cbp* and DSM25430 Δ*cbp* as well as with the corresponding *P. larvae* wild-type strains ATCC9545 and DSM25430. These exposure bioassays were performed essentially as previously described [20], [46], [48]. Briefly, spore suspensions with a defined concentration of colony forming units (cfu) were prepared from each of the four strains to be tested. First-instar larvae were collected from different *Apis mellifera* colonies of the institute's apiary. Larvae were reared in 24-well plates and larval diet (66% royal jelly (v/v), 33% glucose (w/v) and 33% fructose (w/v)) was fed *ad libitum*. During the first 24 h, larval diet of the infection groups was contaminated with spores to achieve infection. Subsequently, larvae were fed with normal larval diet and fresh larval diet was provided every day. Mock infected control larvae fed with normal larval diet during the whole experiment were used as internal quality control of the exposure bioassays. Only assays with less than 15% mortality in the control groups were considered valid. Spore concentrations were identical for the corresponding strains (500 cfu/ml for both ATCC9545 and ATCC9545 Δ*cbp*; 100 cfu/ml for both DSM25430 and DSM25430 Δ*cbp*). The final spore concentrations corresponded to the LC_80_ of the wild-type strains and resulted in 75.5%±5.0% and 77.8%±8.7% mortality in the larvae infected with *P. larvae* ATCC9545 and *P. larvae* DSM25430, respectively, corroborating previous findings [13], [48]. Taking the LC_80_ ensured that observation of both decrease and increase in mortality would be possible. Larval health status and mortality were monitored daily over 15 d. Larvae were only considered to have died from AFB if they contained a high number of vegetative *P. larvae* bacteria after overnight-cultivation of larval remains on CSA plates. *P. larvae* infection was never detected in control animals or in surviving pupae at day 15 post-infection of any of the infection groups. However, vegetative *P. larvae* entrapped in non-degraded PM could be demonstrated in engorged larvae of the *P. larvae* knockout mutant infected groups shortly before defecation. *Cbp*49 knockout stability (presence of the targetron insertion) in *P. larvae* cultivated from larval remains of the knock-out-infected groups was confirmed by PCR with the gene specific primer pair cbp_F and cbp_R (Table 1), flanking the intron insertion site 573/574. PCR amplicons were analyzed by gel electrophoresis on a 1% agarose gel, stained with ethidium bromide and visualised by UV light.

Total mortality of the *P. larvae* wild-type strains and the *P. larvae* knockout mutants was calculated for each replicate as the proportion of larvae that died from AFB compared to the total number of exposed larvae. For each strain, three biological replicates were performed with thirty larvae each. Data represent mean values ± SEM. Data were statistically analyzed by student's t-test.
